# Attribute amnesia is greatly reduced with novel stimuli

**DOI:** 10.7717/peerj.4016

**Published:** 2017-11-14

**Authors:** Weijia Chen, Piers D.L. Howe

**Affiliations:** School of Psychological Sciences, University of Melbourne, Melbourne, Victoria, Australia

**Keywords:** Attribute amnesia, Binding, Attention, Working memory, Expectation, Familiarity

## Abstract

Attribute amnesia is the counterintuitive phenomenon where observers are unable to report a salient aspect of a stimulus (e.g., its colour or its identity) immediately after the stimulus was presented, despite both attending to and processing the stimulus. Almost all previous attribute amnesia studies used highly familiar stimuli. Our study investigated whether attribute amnesia would also occur for unfamiliar stimuli. We conducted four experiments using stimuli that were highly familiar (colours or repeated animal images) or that were unfamiliar to the observers (unique animal images). Our results revealed that attribute amnesia was present for both sets of familiar stimuli, colour (*p* < .001) and repeated animals (*p* = .001); but was greatly attenuated, and possibly eliminated, when the stimuli were unique animals (*p* = .02). Our data shows that attribute amnesia is greatly reduced for novel stimuli.

## Introduction

The degree to which humans remember information once it ceases to be relevant is a contentious topic in cognitive psychology. Some studies have suggested that visual working memory representations are object-based such that attended objects are encoded in their entirety and all their features are stored in memory irrespective of task relevance (e.g., Luck & Vogel, 1997; Luria & Vogel, 2011; Marshall & Bays, 2013). Others dispute this object-based encoding account and argue that observers can remember some features of an object independently of others (e.g., Bays, Wu & Husain, 2011; Fougnie & Alvarez, 2011). In particular, there is evidence that people can selectively encode and maintain a subset of task-relevant features of an attended object and filter out the remaining task-irrelevant features (e.g., Eitam, Yeshurun & Hassan, 2013; Triesch et al., 2003; Woodman & Vogel, 2008). More recently, it has been shown that observers will often be unable to report features of an attended object that are task-relevant but that the observers did not expect to report, a phenomenon referred to as *attribute amnesia* (Chen & Wyble, 2015; Chen & Wyble, 2016). This challenges the common assumption that information in the focus of attention that has reached access awareness (Lamme, 2004) should always be reportable immediately after its presentation.

Following Kanwisher & Driver (1992), attribute amnesia studies have used the term *attribute* broadly to refer to any aspect (e.g., colour, location, identity) of a visual stimulus (Chen & Wyble, 2015; Chen & Wyble, 2016). A typical attribute amnesia experiment starts with an observer performing a number of trials in which they are asked to recall one aspect of a target stimulus before being unexpectedly asked to recall a different aspect of the target stimulus on the “surprise” trial. For example, in Experiment 1 of Chen & Wyble (2016), in each trial the observer was briefly shown a stimulus array comprising four characters, each a different colour. Three of these characters were digits and one was a letter. On the eleven pre-surprise trials, once the stimulus array disappeared the observers were presented with four colours and asked to identify the colour of the letter. On the surprise trial, after the stimulus array disappeared, the observers were, without warning, shown a display containing four letters and asked to indicate which one was the target letter that they had just seen. Although the observers would have attended and processed the target letter when it was presented as part of the stimulus array, most observers were unable to identify the target letter in the test phase of the surprise trial. On the four subsequent trials, i.e., the post-surprise trials, when the observers were now expecting to identify the target letter, they were able to do so. This demonstrates that the amnesia was not due to a limited working memory capacity.

It has long been understood that forgetting can be caused by proactive interference, when previously learned information interferes with the retention of recently learned information (Greenberg & Underwood, 1950; Keppel & Underwood, 1962; Postman & Underwood, 1973). For example, participants who memorised a word list and were tested after two days correctly recalled about 70% of the words; however, those who learned a new list one day after learning the initial list only recalled 40% of the new items. Also, the participants who learned a third list recalled only 25% of it correctly. For those who memorised multiple word lists, the initial list inhibited the learning of new words (Underwood, 1957). Proactive interference was shown to build up across trials and became most prominent when the materials in the prior lists were highly similar to the recent list (Keppel & Underwood, 1962; Underwood, 1957). This is a key feature of attribute amnesia studies where highly similar stimuli are shown on a successive number of trials. Arguably, the failure to recall a key attribute on the surprise trial could reflect a build-up of proactive interference from previously shown stimuli. However, this does not explain why observers were able to recall the same key attribute on the post-surprise trials accurately without a break or a change in the type of memoranda that released them from the built-up interference (Bunting, 2006; Wickens, Born & Allen, 1963).

A common feature of most attribute amnesia experiments is that in the test phase of the surprise trial the distractors are usually highly familiar to the observer. For example, in the experiment described above, there were only four possible letter identities (A, B, D or E), and these were repeatedly shown during the pre-surprise trials. It might be that, in the test phase of the surprise trial, observers have difficulty identifying the target that they have just seen only when the distractor items are also highly familiar to them. Chen & Wyble (2016) acknowledged this possibility and addressed it by performing a second experiment that was identical to the experiment described above except that a different target letter was used on each trial (Chen & Wyble, 2016). As before, they found that participants could not accurately distinguish the target letter from the distractor letters in the test phase of the surprise trial. They concluded that attribute amnesia did not rely on both the target and distractor stimuli being highly familiar due to being repeated across trials. In a subsequent experiment, they found that attribute amnesia was attenuated when observers were required to hold the key attribute (i.e., the one tested in the surprise trial) in memory for 100 ms before using it to identify the target but returned when observers were no longer required to hold it in memory. They concluded that attribute amnesia is due to a failure to consolidate the key attribute into working memory.

While consolidation clearly does reduce attribute amnesia, it remains unclear whether or not attribute amnesia occurs when the distractor stimuli are truly unfamiliar. We use the term *familiar* to denote stimuli that the participants would see repeatedly in their everyday life or during the course of the experiment. For these stimuli, observers possess a conceptual, lexical, or visual representation prior to the surprise trial. Letters are highly familiar stimuli to adult observers due to extensive studying and repeated exposure in everyday life. So, while the findings of Chen & Wyble (2016) rule out the possibility that target repetition per se as the cause of attribute amnesia, it could still be that attribute amnesia occurs only when the distractors in the test phase of the surprise trial are highly familiar.

It has been suggested that observers automatically form a long-term memory trace of any image that they see (Oberauer, 2002), regardless of whether doing so is required for the task. Crucially, we assume that this automatic memory trace allows observers to determine if they had seen an item before, but not necessarily when they have seen the item before. If novel stimuli were to be used on each trial, this would mean that in the test phase of the surprise trial, the three distractors would be novel and the target would be the only item the observer had seen before, having seen it earlier that trial. Thus, the observer could distinguish the target from the distractors based purely on familiarity. Conversely, if all the items are highly familiar, it would not be enough for the observer to know that they had previously seen an item. Instead, they would need to know on which trial they had last seen each item. In particular, in the test phase of the surprise trial, the observer would be highly familiar with both the target and the three distractors, so would not be able to distinguish the target from the distractors based on familiarity. Instead, to determine which of the four items was the target, the observer would need to remember *when* they last saw each item. Thus, the task would be more difficult with familiar stimuli. In summary, we suspect that the observers are well aware of which items they have seen before and only experience difficulty in knowing exactly when they saw each item. Consequently, we predicted that attribute amnesia should not occur for experiments that use novel stimuli on each trial. Our study was designed to test this hypothesis.

## Method

### Participants

Similar studies have established that significant effects can be observed with a sample size of 20 participants (Chen & Wyble, 2015; Chen & Wyble, 2016). We therefore used a minimum sample size of 20 in the first three experiments listed below. In the fourth experiment, we increased the sample size to 40 for reasons discussed later. Each experiment used a different set of participants. In total, 103 students from The University of Melbourne aged between 17 and 50 years (*M* age = 23 years, *SD* = 4.9 years; 30 men) participated. All participants had normal or corrected-to-normal visual acuity (at least 20/25; near-field Snellen eye chart) and normal colour vision (Ishihara plates). This study was approved by The University of Melbourne Human Research Ethics Committee (Ethics ID 1545993.1). All participants gave written informed consent and were remunerated $12 in exchange for their participation.

### Apparatus and stimuli

The stimuli were presented on a personal computer using MATLAB^®^ and the Psychophysics Toolbox (Brainard & Vision, 1997; Pelli, 1997). The target in Experiment 1 was a capital letter in one of the four colours, red (CIE coordinates *x* = 0.60, *y* = 0.35, luminance = 18.5 cd/m^2^), yellow (CIE coordinates *x* = 0.41, *y* = 0.50, luminance = 65.5 cd/m^2^), blue (CIE coordinates *x* = 0.15, *y* = 0.09, luminance = 6.1 cd/m^2^), and magenta (CIE coordinates *x* = 0.34, y = 0.20, luminance = 23.6 cd/m^2^). The distractors were three black capital letters. There were 15 possible letters for the target and the distractors (A, B, C, D, F, H, J, K, L, N, P, R, T, V, and X) and no letter was repeated in the same stimulus array. The stimuli in Experiments 2–4 used colour images of animals and non-animal objects drawn from the image set used in the memory study by Brady et al. (2008). Our participants would not have seen any of these images prior to the experiment. While they may have previously seen images representing some of the same types of objects, as Brady et al. (2008) showed, observers are very good at distinguishing whether or not they have seen different images of the same type of objects or even different images of the same object. As such, we can be confident that all the images used in our study would have seemed novel to our participants. In Experiment 2, four possible animals and four possible non-animal objects were used for all trials. Experiments 3 and 4 were identical to Experiment 2 except that novel images were used in each trial. In each trial, the target was randomly selected from a pool of 200 animal images; and the distractors were randomly selected from a pool of 600 non-animal object images. A mask comprising lines of random length, colour, and orientation was used to mask the stimulus presentation in all experiments.

### Procedure

**Figure 1:**
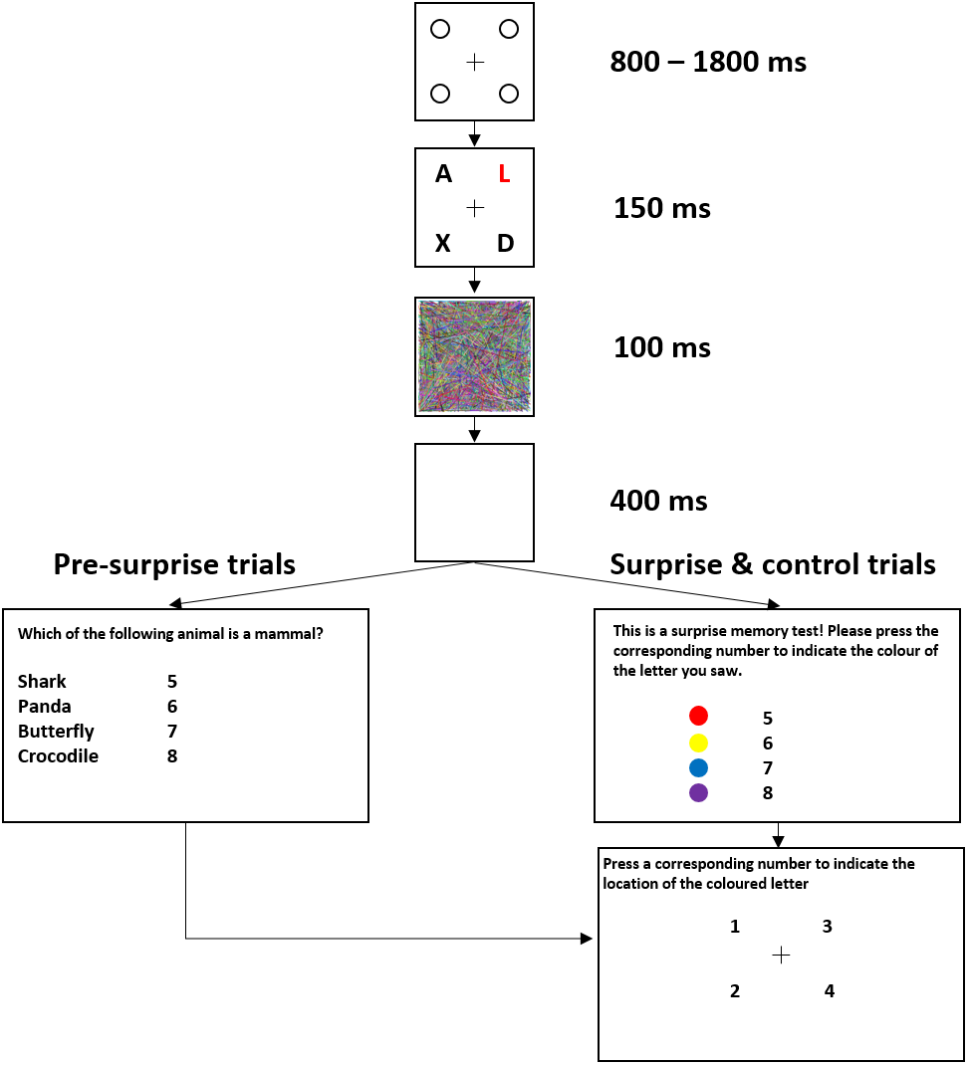
Example of a typical trial sequence for Experiment 1. The colours used were red, yellow, blue, and magenta. The filler question was “Which of the following animals is a mammal?”. In the surprise memory test, the observers were shown four colours and asked to “press the corresponding number to indicate the colour of the target”.

**Figure 2:**
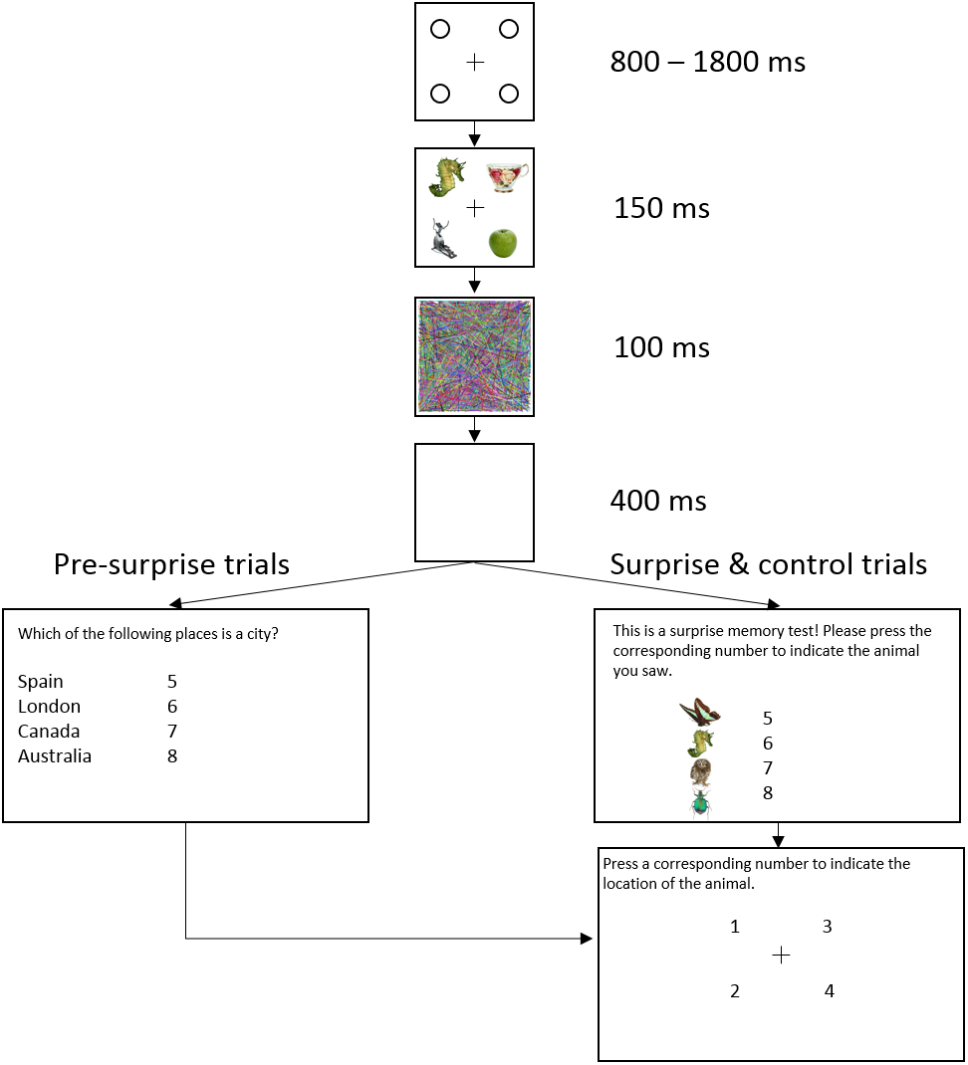
Example of a typical trial sequence in Experiments 2–4. The target was an animal image and the distractors were non-animal images. The filler question was “Which of the following places is a city?” so as not to prime participants to rehearse the identity of the animal. In the surprise memory test, the observers were shown four animal images and asked to “press the corresponding number to indicate the location of the animal”.

Figure 1 shows the trial sequence in Experiment 1. Figure 2 shows the trial sequence for Experiments 2–4. A black fixation cross (0.76° × 0.76°) was shown at the start of each trial centred among four black placeholder circles (1.15° × 1.15°) on a white background. The four placeholders were located at the four corners of a virtual square centred on the screen, subtending an area of 9.62° × 9.62°. This fixation display remained on screen for 800 ms–1,800 ms. Participants were then presented with the stimulus array for 150 ms, which was replaced by the mask for 100 ms and followed by a 400 ms blank screen. For the first 155 trials, a task-irrelevant filler question then appeared on the screen and participants responded by pressing one of four number keys (5–8) to indicate which of the four named animals was a mammal (Experiment 1) or which of the four named places was a city (Experiments 2–4). They were then shown four numbers at the location of the four placeholders and asked to press the corresponding number key to indicate the location of the target, the coloured letter (Experiment 1) or the image of the animal (Experiments 2–4). On the 156th trial, participants were not presented with the filler task but instead, unexpectedly, saw four coloured discs (Experiment 1) or four animal images (Experiments 2–4) with four corresponding numbers (5–8), and were instructed to press the number key corresponding to the target identity. They were then asked to indicate the location of the target in the same way as in the pre-surprise trials. Subsequently, participants received four more post-surprise trials identical to the surprise trial.

## Results

The memory accuracy for location and key attributes (colour in Experiment 1, animal identity in Experiments 2–4) in the four experiments are shown in Table 1. Accuracy in location recall was high in the pre-surprise, surprise, and post-surprise control trials for all four experiments, demonstrating accurate memory for the response attribute (i.e., the attribute that observers expected to report in the pre-surprise trials).

**Table 1:** Memory accuracy for the location and the key attribute (colour in Experiment 1, animal identity in Experiments 2–4) of the target in Experiments 1–4.

	Pre-surprise	Surprise	Control 1	Control 2	Control 3	Control 4
Location						
Exp. 1	0.93	0.76	0.86	0.90	0.90	0.90
Exp. 2	0.97	0.86	0.95	1.00	1.00	1.00
Exp. 3	0.93	0.90	0.95	1.00	1.00	1.00
Exp. 4	0.95	0.95	0.93	0.98	0.93	0.95
Colour/Animal						
Exp. 1	N/A	0.48	1.00	1.00	0.95	1.00
Exp. 2	N/A	0.45	0.91	1.00	1.00	1.00
Exp. 3	N/A	0.80	0.95	1.00	1.00	1.00
Exp. 4	N/A	0.90	0.93	1.00	0.93	0.95

Attribute amnesia was observed in Experiments 1 and 2. Only 10 out of 21 participants correctly reported the colour of the target letter in Experiment 1 in the surprise trial; and 10 out of 22 participants correctly reported the identity of the repeated-animal target in Experiment 2 in the surprise trial. Performance improved significantly in both experiments on the first control trial (colour: 100% vs 48%, *χ*^2^[1, *N* = 42] = 14.9, *p* < .001, *ϕ* = .60; repeated-animal: 91% vs 45%, *χ*^2^[1, *N* = 44] = 10.5, *p* = .001, *ϕ* = .49). Recall accuracy for both colour and repeated-animal identity remained high in the three subsequent control trials. However, participants performed well in the surprise trial in Experiment 3, 16 out of 20 participants correctly recognised the unique-animal. The difference between unique-animal identity recall in the surprise trial and the first control trial was non-significant, *χ*^2^(1, *N* = 40) = 2.06, *p* = .15, *φ* = .23.

In order to confirm these findings with greater statistical power, we re-run Experiment 3 with 40 participants (Experiment 4) and observed the same pattern of results. In this confirmatory experiment, 36 out of 40 participants correctly identified the target animal in the surprise trial; this accuracy was not significantly lower than the memory accuracy in the subsequent control trial, *χ*^2^(1, *N* = 80) = 0.16, *p* = .69, *ϕ* = .04.

**Figure 3:**
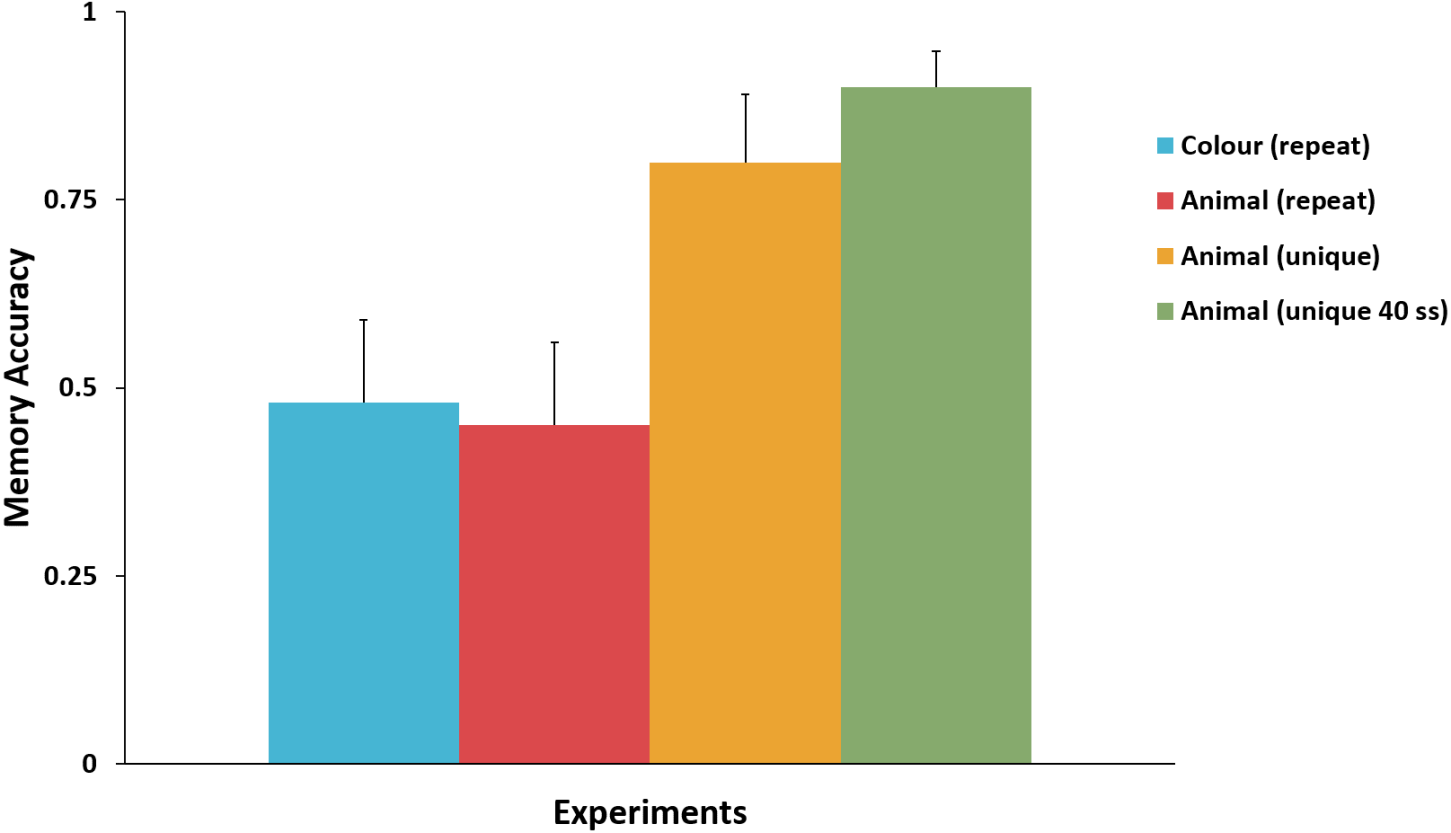
Comparison of the memory accuracy for the key attribute in the surprise trial across Experiments 1–4. Memory accuracy for unique-animal identity was significantly higher than that for repeated-animal identity and colour. No difference in memory accuracy was observed between colour and repeated-animal identity, or between the two unique-animal identity experiments. Error bars represent + SEM.

Figure 3 presents the mean accuracy for the key attribute in the surprise trial in Experiments 1–4. There was no significant difference between mean memory accuracy in Experiment 1 (colour) and Experiment 2 (repeated-animal identity), *χ*^2^(1, *N* = 43) = 0.02, *p* = .89, *ϕ* = .02. Nor was there a significant difference between mean memory accuracy between Experiments 3 and 4, *χ*^2^(1, *N* = 60) = 1.15, *p* = .28, *ϕ* = .14. Comparing Experiment 3 to Experiments 1 and 2 we found that mean memory accuracy for the unique-animal identity was significantly higher than the mean memory accuracy for both repeated-animal identity, *χ*^2^(1, *N* = 42) = 5.30, *p* = .02, *ϕ* = .36; and colour, *χ*^2^(1, *N* = 41) = 4.63, *p* = .03, *ϕ* = .34. Comparing Experiment 4 to Experiments 1 and 2 produced an equivalent result: mean memory accuracy for the unique-animal identity was significantly higher than the mean memory accuracy for both colour, *χ*^2^(1, *N* = 61) = 13.34, *p* < .001, *ϕ* = .47, and repeated-animal identity, *χ*^2^(1, *N* = 62) = 14.71, *p* < .001, *ϕ* = .49.

## Discussion

Our experiments have shown that attribute amnesia is greatly reduced, and possibly even eliminated, when the distractors are novel. In Experiments 3 and 4, each trial used a novel animal image as the target item. On the surprise trial, observers were very good at distinguishing the target that they had just seen from three novel distractor images. Experiment 2 used an identical procedure except that only four animal images were used for all 155 pre-surprise trials. Thus, the observers became highly familiar with these images. For this experiment, on the surprise trial the observers had difficulty distinguishing the target image from the distractor images. This finding was not unique to the animal images used in our experiment. An essentially identical finding occurred in Experiment 1, which used conventional letter stimuli, similar to those used previously, and replicated these previous findings (Chen & Wyble, 2015; Chen & Wyble, 2016).

Our finding that attribute amnesia is greatly reduced when the distractor items in the test phase of the surprise trial are novel constrains explanations of the phenomenon. For example, this finding contradicts the consolidation theory of attribute amnesia proposed by Chen & Wyble (2016). Consolidation theory does not take into account item familiarity so cannot explain why attribute amnesia would occur in Experiment 2 but not in Experiments 3 and 4 as the only difference between Experiment 2 and Experiments 3 and 4 was that the targets in Experiments 3 and 4 were unfamiliar to the subjects.

Our findings might represent a form of proactive interference. The lower recall accuracy for a repeated animal compared to a unique may have been due to the higher intra-list similarity causing a greater build-up of interference. However, a recent study challenges this explanation. Chen, Swan & Wyble (2016) asked participants to track a coloured ball passing between six players for 5–59 s. In the first 31 trials, participants reported the number of passes. On the 32nd trial, they were unexpectedly quizzed about the colour of the ball and 37% of them reported a wrong colour. The error rate dropped to 17% and stayed low in the subsequent four control trials. Conversely, when observers in a control experiment were asked to report the colour of the ball on the first trial, their error rate was only 20% and stayed consistently low over the next 30 trials. If observers were unable to correctly report the colour on the surprise trial due to a build-up of interference over the preceding 31 trials, a similar level of interference should be observed in the 30 “post-surprise” trials in the control experiment. But this was not the case. Therefore, we believe that the failure to unexpectedly recall a key attribute that was previously in the focus of attention cannot be entirely due to proactive interference.

So why would attribute amnesia be reduced for unfamiliar stimuli? We suggest that the reason for this is that observers are able to recall what they have seen before but have difficulties in recalling exactly when they have seen these items/attributes before. This means that when the distractors in the test phase of the surprise trial are novel, observers can readily identify the target item as it is the only item they have seen before. This would explain why we observed a marked reduction in the attribute amnesia phenomenon in Experiments 3 and 4 since in those experiments the distractors in the test phase of the surprise trial were all novel. Conversely, when the distractors in the test phase of the surprise trial have all been targets on previous trials, to identify the target on that trial, the observers must recall on which trials the items had been targets. If the observers have difficulty recalling exactly when they last saw each item/attribute they will have difficulty performing the task. This would explain why we found a strong attribute amnesia effect in Experiments 1 and 2 since in those experiments all the distractors in the test phase of the surprise trial had been shown previously.

In summary, Experiments 3 and 4 show that, on the surprise trial, the observers had little difficulty indicating which image they had seen before from a collection of four images, three of which were completely novel. Conversely, Experiments 1 and 2 show that when presented with four images all of which had been shown before multiple times, observers had great difficulty in recalling which image had appeared on that particular trial. In other words, attribute amnesia is not a total amnesia: observers are not completely unaware of which images they had seen before. Rather, attribute amnesia is much more limited: it is the inability to distinguish which highly familiar image was presented on that particular trial, when observers are not expecting to do this task. However, observers can do this task when they expect to do it. In Experiments 1 and 2, after the surprise trial, observers were very accurate at indicating which of the four colours (Experiment 1) or animal images (Experiment 2) had been the target on each trial.

These results suggest that memory for whether or not an image has previously been shown is automatic but memory for whether or not the image has been shown on that particular trial is not. This suggests that observers automatically form a long-term memory trace of any image that they see (Oberauer, 2002), regardless of whether doing so is required for the task. However, it seems that observers only “bind” this long-term memory trace to the current trial if the task requires them to do so, a proposal known as the expectation-based binding hypothesis (Chen, Swan & Wyble, 2016).

The expectation-based binding hypothesis can also explain the findings of Jiang et al. (2016). In their experiments, observers were shown four numbers in each trial. For half the observers, three of the numbers were even and one was odd. For these observers, the task was to identify the location of the odd number. For the remaining observers, three of the numbers were odd and one was even. For these observers, the task was to identify the location of the even number. On the surprise trial, observers were presented with four numbers and asked to identify the target of that trial. Observers were very poor at doing this, exhibiting clear attribute amnesia. Despite this, it was found that on the pre-surprise trials observers were more accurate and faster at identifying the location of the target number when the target number was repeated from the previous trial. The authors took this as evidence that, despite the attribute amnesia, the target number had been memorised. This is consistent with the expectation-based binding hypothesis that also claims that the target item is always memorised whether or not binding occurs.

The findings in this study seems almost contradictory to the research into familiarity in the context of multiple identity tracking (MIT) where both the location and identity of repeated targets were easier to maintain than random targets (Pinto et al., 2010). When observers tracked the same four cartoon animals on every trial, they could identify their identities more accurately than when they tracked a different set of animals on each trial. But this repetition benefit was reduced when the targets were masked during tracking. This was taken as evidence that target repetition did not merely reduce the memory load by making targets easier to encode into memory, but improved identity tracking by improving the maintenance and recognition of identities, or making identity-location binding easier (Pinto et al., 2010). Nonetheless, there is a noticeable difference between our study and studies of MIT. Participants need to solve the binding problem in order to track the location and identity of a moving object, but they do not expect to bind target identity with its trial prior to the surprise trial in our experiments. It may well be that familiarity or repetition improves identity-location binding, but unless the participant expects to report both attributes, such binding does not occur.

Our results also speak to the broader issue of how objects are encoded in memory. According to the feature-based encoding hypothesis of object representation, attributes of an attended object are selectively encoded into working memory based on their task-relevance (Serences et al., 2009; Woodman & Vogel, 2008). According to this hypothesis, features crucial to the selection of a target are clearly relevant and should therefore be reportable. The data from our first two experiments contradicts this prediction. Alternatively, if feature encoding was based on response-relevance and not task-relevance, this hypothesis still cannot explain how most observers can accurately recognise the identity of a unique animal that they did not expect to report as was demonstrated in Experiments 3 and 4. As such, the feature-based encoding hypothesis does not seem to be compatible with our data.

As an alternative to the feature-based encoding hypothesis, the object-based encoding hypothesis assumes that all features of an attended stimulus are stored in working memory as an integrated object, regardless of whether they are relevant or not (Gao et al., 2016; Luria & Vogel, 2011; Marshall & Bays, 2013). An inability to report a key attribute of the target in this and previous research (Chen & Wyble, 2015; Chen & Wyble, 2016; Jiang et al., 2016) challenges this hypothesis. Even a weaker hybrid model, where encoding for an entire object only occurs when working memory load is not exceeded, cannot fully account for our results. In both Experiments 1 and 2 where observers failed to report the key attribute in the surprise trial, they needed to attend to only a single object which should be well within the working memory capacity. Furthermore, any claim that this single object was somehow sufficiently complex that it exceeded the memory capacity then has difficulty explaining how observers were able to do the task in Experiments 3 and 4. Taken together, Experiments 1–4 strongly argue against this hypothesis.

Our data is more consistent with the hierarchical feature bundle model proposed by Brady, Konkle & Alvarez (2011) where the “unit” of visual working memory is a hierarchical hybrid of an integrated object representation at the top level supported by individual low-level feature representations at the bottom. The initial encoding process is object-based but the lower level features of an attended object allows for differential modulation of the fidelity of features independently, so some features are processed more elaboratively while the encoding of others can be fragile depending on whether they are task relevant (Brady, Konkle & Alvarez, 2011). Observers expected to report location information but not identity information. This difference in expectation may have led to *differential rehearsal* of the features so that the target location was rehearsed and kept active in working memory until a response was made, but the target identity received minimal rehearsal (Basden, Basden & Gargano, 1993; Mitchell et al., 2002). This would have made the latter susceptible to interference from familiar distractors when observers were unexpectedly asked to report the target identity, and could only respond accurately when the distractors were novel.

In conclusion, our findings impose an important limit on attribute amnesia: it occurs only when the distractor stimuli in the test phase of the surprise trial are familiar. This finding contradicts the consolidation theory of attribute amnesia and cannot be fully accounted for by proactive interference. It is, however, consistent with both the expectation-based binding hypothesis of Chen, Swan & Wyble (2016) and the hierarchical feature bundle model of Brady, Konkle & Alvarez (2011). Although attribute amnesia is a very robust laboratory finding, it doesn’t seem to occur as readily in real life. Our results speak to this issue. Humans are able to rapidly consolidate into long-term memory large number of images depicting natural objects (Brady et al., 2008). Furthermore, these images are consolidated with high fidelity in that observers are readily able to distinguish between these images and highly similar images that they had not seen before. For example, observers can even notice when the state of the object is changed in some minor way (Brady et al., 2008). This means that for attribute amnesia to occur in real life the observer would need to be repeatedly shown the exact same objects in the exact same states. In practice, this is a rare occurrence, which could explain why attribute amnesia rarely occurs in the real world.

##  Supplemental Information

10.7717/peerj.4016/supp-1 Data S1 Raw data of response correctness for target location and the surprise question in Experiments 1–3

## Additional Information and Declarations

### Data Availability

The following information was supplied regarding data availability: The raw data has been supplied as a Supplemental File.
